# Introducing oral tobacco for tobacco harm reduction: what are the main obstacles?

**DOI:** 10.1186/1477-7517-4-17

**Published:** 2007-11-07

**Authors:** Yves Martinet, Abraham Bohadana, Karl Fagerström

**Affiliations:** 1 Unité de Tabacologie, Service de Pneumologie, Centre Hospitalier Universitaire, Nancy, France; 2 INSERM U724, Université Henri Poincaré, Nancy, France; 3 INSERM ERI 11, Vandoeuvre-lès-Nancy, France; 4 Smoker Information Centre, Helsingborg, Sweden

## Abstract

With the number of smokers worldwide currently on the rise, the regular failure of smokers to give up their tobacco addiction, the direct role of smoke (and, to a much lesser extent, nicotine) in most tobacco-related diseases, and the availability of less toxic (but still addictive) oral tobacco products, the use of oral tobacco in lieu of smoking for tobacco harm reduction (HR) merits assessment.

Instead of focusing on the activity itself, HR focuses on the risks related to the activity. Currently, tobacco HR is controversial, generally not discussed, and consequently, poorly evaluated.

In this paper, we try to pinpoint some of the main reasons for this lack of interest or reluctance to carry out or fund this type of research. In this paper we deal with the following issues: the status of nicotine in society, the reluctance of the mainstream anti-tobacco lobby toward the HR approach, the absence of smokers from the debate, the lack of information disseminated to the general population and politicians, the need to protect young people, the role of physicians, the future of HR research, and the role of tobacco companies.

## 1. Introduction

The leading avoidable cause of death worldwide, tobacco smoking [1] is due to an addiction to tobacco [2]. Tobacco is a popular and legal commodity, as well as caffeine and alcohol, commercialized by a handful of extremely powerful transnational tobacco corporations. Despite major efforts by the "health community" to curb the so-called "tobacco epidemic," it is likely to remain, along with alcohol, one of the most popular psychoactive drugs for the next several generations. Although tobacco contains other substances besides nicotine that likely contribute to its pleasure and addiction, nicotine is necessary for the strong addictive power of tobacco [3,4].

Today's key tobacco control policies are based on supply and demand reduction strategies [1], as reflected in the World Health Organization's (WHO) Framework Convention on Tobacco Control (FCTC), currently ratified by over 130 countries [5].

Obviously, it is mandatory for countries ratifying the FCTC to implement its provisions. However, even countries with strong, effective regulatory policies and smoking cessation clinics in place must still deal with a significant number of continuing smokers, as well as newly recruited young smokers. More importantly, most countries with poor regulatory policies and tobacco cessation programs are characterized by an increase in the number of smokers, mainly due to a sharp rise in female smoking [6].

As a result, hundreds of millions of human beings worldwide smoke tobacco every day. Most smokers, sooner or later, will try to give up their deadly addiction. Unfortunately, due to nicotine's strong addictive power, the vast majority of these smokers will fail, even after several attempts, and, eventually, a great number of them will die from smoking-related diseases [5].

Despite the limited efficiency of current treatments for tobacco addiction, most academic and medical recommendations are based on abstinence. The ultimate goal of this "quit or die" approach [7] is total eradication of nicotine/tobacco use. However, numerous studies have shown that while smokers smoke to a large extent for nicotine self-administration, most do not die from nicotine itself, but rather from inhaling a complex smoke made of a mixture of more than 4000 products [8]. Interestingly, smoking is not the only way to self-administer tobacco nicotine. In this respect, the use of smokeless tobacco, mainly oral tobacco (including Swedish snus), is particularly interesting, since oral tobacco use has been convincingly shown to be much less harmful than cigarette smoking [9,10]. Conservative estimates suggest a ratio of oral tobacco use risk *vs*. tobacco smoking risk of about 1–5/100 [11]. In this respect, among Swedish men–with a relatively high prevalence of daily nicotine use of about 32%–only 13% smoke and 22% use snus [12], which could be a reason for the very low incidence of lung cancer in this population [13,14]. Furthermore, smokers use oral tobacco as frequently as nicotine replacement products as a first step to quit smoking and ultimately nicotine [15].

Thus, in light of these four main facts: (1) the high number of smokers worldwide, (2) the regular failure of smokers to give up their tobacco addiction, (3) the direct role of several smoke components, and, to a much lesser extent, nicotine, in most tobacco-related diseases, and (4) the possible use of much less toxic, but still addictive, tobacco products, evaluation of less harmful products, such as oral tobacco, for the purpose of harm reduction is warranted. Although this proposal may sound reasonable, it currently faces strong opposition [16].

This issue is poorly debated within the anti-tobacco lobby, with some questions being considered almost taboo. In contrast, tobacco companies are markedly active in this field, and this discrepancy of interest will eventually put pressure on politicians to decide on the issue, in the absence of any real popular debate. This paper will discuss the main factors contributing to this situation, and will ask some central questions, the answers to which should be based on scientific evidence, rationality, and respect for human rights.

## 2. Discussion

### The societal impact of nicotine

Whatever the reasons may be, there is no known human society whose members or citizens do not use at least one legal psychoactive substance [17]. Social tolerance of a specific product by a given people is mainly based on traditions, which explains why a product's use may be legal in one country but not in another [17]. Tobacco use is legal in almost all countries, for several reasons: the tobacco plant is quite easy to grow almost anywhere, its use is convenient, and it doesn't alter users' judgment or ability to work. But tobacco use is also very popular as a result of the tobacco industry's remarkable efficiency in promoting its sale, using all possible legal and illegal means [18].

The terrible health consequences of tobacco smoking being largely known, it is important to understand why people still smoke, even if it has been suggested that the health benefit of smoking cessation may partially be offset by the weight gains. Obviously, nicotine addiction plays a central role, but tobacco users' expectations from nicotine and the impact of tobacco product marketing should also be taken into account. For some psychiatric patients (schizophrenia, depression...) tobacco has been suggested to be useful as a self-medication, although the possibility exists that it may contribute to the occurrence of some psychiatric symptoms [19-21]. Interestingly, most smokers use nicotine for its psychoactive properties: brain stimulant, helping users focus attention, relaxant, and appetite suppressant. Furthermore, self-administration of nicotine induces a feeling of pleasure, contributing to its recreational use, while cigarette sharing is part of its social acceptability.

In view of the popularity and the addictive nature of tobacco use, one can ask the following questions in respect to the place that this psychoactive drug could/should have in our society: Assuming that the harm related to nicotine use could be reduced to a level acceptable (to be defined) by its users and society, what status should be attributed to nicotine among other psychoactive drugs? Should recreational use of nicotine be definitively prohibited? In other words, are we heading for a nicotine-free world, or, at least as a first pragmatic step, a low risk nicotine use world?

### Harm reduction in addiction control

Harm reduction (HR) is a general concept stating that, when it is not possible to forbid/eradicate a risky human activity, the best alternative is to try, to the extent possible, to reduce its harm. The concept has been applied in a variety of situations, including various road safety policies, needle exchange programs for intravenous drug users, even the use of abseiling ropes and helmets for climbing. Instead of concentrating on the activity itself, HR focuses on the risks related to the activity. In the addiction field, several medical trials of prescription heroin, plus methadone maintenance treatment for long-term heroin users are currently underway in several countries (Switzerland, Netherlands, UK, Canada, USA). It is unfortunate that research that appears to be sound for heroin, an illegal drug, has yet to be conducted for tobacco, a legal drug.

Currently, most opposition to tobacco HR comes from the mainstream anti-tobacco lobby, rather than the tobacco industry. Year 2006 WHO Tobacco Free Initiative (TFI) World No Tobacco Day slogan, "Tobacco: deadly in any form or disguise" [16], exemplifies the lobby's resistance to HR.

We, and others [22-25], believe that, if the WHO supply and demand approach is to be backed as a solid base for building up a strong worldwide anti-tobacco policy, then tobacco HR should also be evaluated and/or promoted within a hierarchy of "achievable" goals. The fact that a first attempt to implement HR strategy, using so-called "low tar, low nicotine" cigarettes, failed [26] should not discourage investigators from evaluating oral tobacco for HR, since most epidemiological observations confirm its low toxicity compared to tobacco smoking [27-30].

Considering the poor efficiency of tobacco addiction treatments and the possible alternate use of less toxic tobacco products instead of cigarettes, it is legitimate to ask why does the mainstream anti-tobacco lobby shun the idea of evaluating oral tobacco use for tobacco HR? What is more important, pragmatism or dogmatism?

### Tobacco smokers are the problem

Whereas users of other drugs are relatively well represented in their respective drug addiction NGOs, tobacco users are very poorly represented within anti-tobacco NGOs. The lack of smoker members of anti-tobacco lobbies is surprising, since tobacco use is by far the most common addiction, and is the number one killer with respect to drug use. Given the central role played by other drug user NGOs in promoting HR, the absence of any structured smokers' lobby (with the exception of the pro-tobacco lobby) may explain, at least in part, current negative perceptions of tobacco HR. It may also reflect overall consumer ignorance about the relative toxicities of the various forms of medicinal and tobacco nicotine. Furthermore, in Europe and North America, tobacco smokers, currently representing 15–30% of the adult population, are almost never directly involved in formulating policies addressing their chronic disease, as tobacco addiction is currently defined. In contrast, individuals with other chronic diseases, including diabetes, cystic fibrosis and cancer are much more organized and proactive with respect to policy formulation.

This absence of smoker involvement probably stems from the "legal" status of tobacco, but also from its widespread use, and from smokers' ambivalence about their status. On one hand, they usually know that they are tobacco-dependent, but, on the other hand, they often like to see themselves as free, and responsible for their personal lifestyle choices. The tobacco industry plays a major role in this illusion [31].

Even though the "low tar, low nicotine" cigarette experience has been, as far as actual harm reduction is concerned, a major failure, the widespread commercial success of these cigarettes suggests that, given a choice, smokers would change their smoking habits as part of personal HR strategy. Obviously, the tobacco industry contributed to this failure by hiding its knowledge about compensatory smoking [31].

Given the absence of involvement of tobacco users in Tobacco Control, in marked contrast to the illicit drugs use field, the two following questions are obvious: If smokers are the problem, why aren't they also part of the solution? Shouldn't they be involved in planning tobacco-related policies? Is it ethical to keep them unaware of these issues?

### The general population

The direct and indirect costs of tobacco smoking are huge [32], not to mention human suffering. Surprisingly, despite the regular action of numerous "anti-tobacco" NGOs (some defined by very narrow interests limited to protecting their members from passive smoking or preventing smoking uptake by adolescents), most of the general population is totally ignorant of the possibilities of tobacco HR. There seems to be little interest among NGOs to involve or learn from smokers, particularly smokers who do not want to stop. Since promoting HR would result in a significant decrease in health costs, laypersons should get sound, clear, and credible information about the risks/advantages of each nicotine-containing product.

The huge health burden of tobacco use on the society justifies that tobacco HR should be debated in public as a social issue. Isn't the economic burden of tobacco a sufficient reason to justify it?

### Young people should be protected

Since giving up smoking is very difficult, significant efforts should be focused on preventing children from starting to use tobacco products. However, easy oral tobacco availability, associated with the likely presumption that "safer" means "safe", may lead to an increase in the number of oral tobacco users. Furthermore, it has been suggested that oral tobacco use could be a gateway to tobacco smoking [33]. This central issue needs to be seriously addressed. Indeed, if promotion of cigarette smoking HR results in a significant increase of new smokers, this policy would be a failure. Currently, the bulk of the data from studies best addressing this topic shows that use of smokeless tobacco protects from later smoking, i.e. more would have started smoking without smokeless tobacco than would have switched to smoking after starting with smokeless tobacco [15]. Nevertheless, close monitoring of this possible gateway effect should be carried out in case an HR policy based on oral tobacco use is implemented. Finally, since cannabis is usually smoked with tobacco, reducing tobacco smoking in the general population may contribute to a decrease of cannabis use.

### Physicians should get involved

Most physician training in smoking cessation is quite recent [34], and is mainly based on medicinal nicotine and the "quit or die" dogma [7]. However, while medicinal nicotine can reduce cigarette craving to some extent, it fails to provide some smokers with the "fix" they miss so badly, and this explains, at least in part, the high relapse rate. Quite recently, physicians in some countries have been allowed to prescribe medicinal nicotine (nicotine replacement therapy, NRT) for HR, with a concomitant reduction of the number of cigarettes smoked daily. However, long-term health effects of this type of NRT-based HR are not known: how long will smokers be able to smoke only a few cigarettes a day? How much compensatory smoking [35] is involved? Furthermore, in the case of lung cancer incidence, the number of years a smoker has smoked is much more important than the actual number of cigarettes smoked.

Advising cigarette smokers who cannot give up smoking to use oral tobacco could be an efficient way to reduce harm related to nicotine addiction [36]. Physicians regularly deal with HR issues when making decisions, for example, indicating a mutilating or high-risk surgery for a life threatening disease, prescribing chemotherapy with significant side effects to treat cancer etc.

In this respect, it is more comfortable for a physician to blame a smoker for not being able to give up smoking, despite the best current medical treatments. Wouldn't it be more ethical to encourage some smokers to switch to oral tobacco as a HR strategy?

### Lawmakers should get involved

With respect to psychoactive drug use, the policy-maker's position is not always an easy one. She/he could be accused of being either too liberal or not liberal enough. Personal and/or family history *vis-à-vis *drug use may affect the lawmaker's ability to make fair decisions. Nevertheless, given the cost of smoking to society, public officials should at least consider tobacco HR strategies. For example, the EU ban on snus sales outside Sweden is a central issue. While it is difficult to officially advise the population to use a specific tobacco product, it might be possible to apply a tobacco tax that is proportionate to each product's degree of harm. Moreover, since smokers are more often found among poorer populations, such a policy would be both efficient and socially fair.

Even if it is a delicate issue with major direct and indirect implications, shouldn't lawmakers support a comprehensive global policy on nicotine addiction, including HR?

### Promoting research on HR

Research on the health effects of new tobacco products is difficult, since investigators must wait 10–20 years before the full health impact of these products is observed. To date, there are no reliable surrogate biomarkers available to predict tobacco-related disease risk. Such tools are urgently needed to circumvent the long waiting period for data [37]. Furthermore, under current ethical guidelines, large scale, long-term prospective studies would be difficult to carry out. Thus, in light of HR's ultimate goal, the most efficient strategy remains promotion of total abstinence from smoking. Unfortunately, only tobacco products, not NRT, can currently provide a "fix", and, among these, oral tobacco products are the least harmful. However, even in countries where oral tobacco is freely available on the market, not all smokers will switch to a less harmful product. In Sweden, for example, 13 % of men and 18% of women still smoke, while 22 % of men and only 3% of women use snus. Thus, individual acceptability of oral tobacco products should be evaluated in each country and for various groups of smokers, since it may vary considerably.

Pharmaceutical companies should carry out research on new medicinal nicotine- delivery devices mimicking the effects of cigarettes as much as possible, including the ability to induce a fix. Of course, this research may ultimately lead these companies to sell "addictive medicinal nicotine" products. However, this should not be perceived as an obstacle, *per se*, since they already sell addictive products such as morphine, heroin (discussed above), and tetrahydrocannabinol in some countries. This research should be funded by the pharmaceutical industry and carried out in accordance with current medical research standards.

With respect to research on less harmful oral tobacco products, given the tobacco industry's poor ethical record [31], a closely monitored experimental setting should be designed to secure total independence from the tobacco industry. Since the health, social and financial implications of such research, including the monitoring of young people's tobacco use, are so great, this research should be carried out under public guidance. Financing, on the other hand, should come from the tobacco industry under conditions forbidding tobacco companies from having any influence on the research carried out. Moreover, researchers should be able to apply for public funds to carry out this type of research. This approach would be in keeping with the current policy of most academic institutions and scientific societies around the world that prohibits research financing by the tobacco industry.

It is the responsibility of the society to work with pharmaceutical companies in respect to the possible marketing of "addictive medicinal nicotine" to smokers. However, is our society ready to allow its use by non-smokers for recreational purposes?

### Tobacco companies

Tobacco companies do not promote oral tobacco use for humanitarian purposes, but rather to keep or increase market share and ultimately earn healthy profits. Promoting HR with tobacco products is one way for them to keep as many consumers as possible, including tobacco smokers who want to quit, while projecting the image of a responsible industry that cares for consumers.

However, with time, if new tobacco users are not recruited, the number of cigarette and/or oral tobacco users will inevitably drop. Another fact is that most new customers of any drug are found among young people. Thus, the tobacco industry will target them, directly and indirectly, telling them that compared to other tobacco products, oral tobacco is "safe" (or "almost safe"), and that it is fashionable to use it.

Thus, any responsible public health policy promoting oral tobacco use for tobacco HR should be carried out in a strictly state-controlled manner, requiring: (1) that information about tobacco products be disseminated under regulatory agency control; (2) use of generic packaging; (3) prohibition of sales to individuals under age 18; and (4) forbidding tobacco industry to operate in a free market. In this respect, it could be fruitful to examine the previously adopted regulatory systems for dealing with the possible risks of unintended consequences observed in the pharmaceutical and the beverage alcohol businesses.

It is the responsibility of politicians and public health experts to work on a comprehensive, global Tobacco Control policy including HR with oral tobacco through a tight control of the tobacco industry. In this respect, is it morally acceptable to make profit from selling tobacco?

## 3. Conclusion

Oral tobacco use for tobacco HR, and, more broadly, the status of nicotine within our society should be largely and openly debated. Regardless of the long-term outcome, it is unethical at this time not to evaluate the use of oral tobacco for smokers who cannot give up cigarette smoking, and will die from their addiction. WHO FCTC is a major step toward progressive control of tobacco use. However, the supply/demand approach should not prevent evaluation of other major strategies such as HR. Finally, smokers and the general population should be more clearly involved in the planning of tobacco/nicotine regulatory policy.

## Competing interests

The authors have no conflict of interest to declare. Karl Fagerstrom has consulted for numerous pharmaceutical companies with an interest in treatment of tobacco dependence. He also owns stock in NicoNovum, a company developing nicotine replacement products.

## Authors' contributions

YM, AB, and KF equally contributed to the elaboration of this manuscript. All authors read and approved the final manuscript.

## References

[B1] Esson KM, Leeder SR (2005). The millennium development goals and tobacco control: an opportunity for global partnership.

[B2] The World Health Organization (1992). The ICD-10 classification of mental and behavioural disorders.

[B3] Kunin D, Latendresse MW, Gaskin S, Smith BR, Amit Z (2000). Preexposure effects of nicotine and acetaldehyde on conditioned taste aversion induced by both drugs. Pharmacol Biochem Behav.

[B4] Fowler JS, Logan J, Wang GJ, Volkow ND (2003). Monoamine oxidase and cigarette smoking. Neurotoxicology.

[B5] World Health Organization Updated status of the WHO Framework Convention on Tobacco Control. Available at. http://www.who.int/tobacco/framework/countrylist/en/index.html.

[B6] Shafey O, Dolwick S, Guindon GE (2003). Tobacco control country profiles. Atlanta (GA) USA: American Cancer Society.

[B7] Fiore MC, Bailey WC, Cohen SJ (2000). Treating tobacco use and dependence: clinical practice guideline. Rockville (MD) USA: Department of Health and Human Services Public Health Service.

[B8] Green surDR. surRodgman A (1996). The tobacco chemist's research conference. A half century of advances in analytical methodology of tobacco and its products. Recent Adv Tob Sci.

[B9] Foulds J, Ramström L, Burke M, Fagerström K (2003). Effect of smokeless tobacco (snus) on smoking and public health in Sweden. Tob Control.

[B10] Levy DT, Mumford EA, Cummings KM, Gilpin EA, Giovino G, Hyland A, Sweanor D, Warner KE (2004). The relative risks of a low-nitrosamine smokeless tobacco product compared with smoking cigarettes: estimates of a panel of experts. Cancer Epidemiol Biomarkers Prev.

[B11] Royal College of Physicians (2002). Protecting smokers, saving lives. The case for a tobacco and nicotine regulatory authority.

[B12] http://www.tobaksfakta.org/Default.asp?bhcd=32&bhsh=600&bhsw=800.

[B13] Zatonski W (2003). Lung cancer trends in selected European countries: what we can learn from the Swedish Experience with oral tobacco (snuff). ENSP Status Report on Oral Tobacco.

[B14] Levi F, Lucchini F, Negri E, La Vecchia C (2004). Trends in mortality from major cancers in the European Union, including acceding countries, in 2004. Cancer.

[B15] Ramström LM, Foulds J (2006). Role of snus in initiation and cessation of tobacco smoking in Sweden. Tob Control.

[B16] (2006). Tobacco deadly in any form or disguise.

[B17] Von Gernet A, Ferrence R, Slade J, Room R, Pope M (2001). Origins of nicotine use and the global diffusion of tobacco. Nicotine and Public Health.

[B18] Hurt RD, Robertson CR (1998). Prying open the door to the tobacco industry's secrets about nicotine: the Minnesota Tobacco Trial. JAMA.

[B19] Sacco KA, Termine A, Seyal A, Dudas MM, Vessicchio JC, Krishnan-Sarin S, Jatlow PI, Wexler BE, George TP (2005). Effects of cigarette smoking on spatial working memory and attentional deficits in schizophrenia: involvement of nicotinic receptor mechanisms. Arch Gen Psychiatry.

[B20] Gehricke J-G, Wahlen CK, Janner LJ, Wigal TL, Steinhoff K (2006). The reinforcing effects of nicotine and stimulant medication in the everyday lives of adult smokers with ADHD: A preliminary examination. Nicotine Tob Res.

[B21] Salin-Pascual RJ (2002). Relationship between mood improvement and sleep changes with acute nicotine administration in non-smoking major depressed patients. Rev Invest Clin.

[B22] Gray N, Henningfeld J, Benowitz NL, Connolly GN, Dresler C, Fagertrom K, Jarvis MJ, Boyle P (2005). Toward a comprehensive long term nicotine policy. Tob Control.

[B23] Crane J, Blakely T, Hill S (2004). Time for major roadworks on the tobacco road?. NZ Med J.

[B24] Sumner W II (2003). Estimating the health consequences of replacing cigarettes with nicotine inhalers. Tob Control.

[B25] Martinet Y, Bohadana A, Fagerström K (2006). Would alternate tobacco products use be better than smoking. Lung Cancer.

[B26] Shiffman S, Pillitteri JL, Burton SL, Rohay JM, Gitchell JG (2001). Effect of health messages about "Light" and "Ultra Light" cigarettes on beliefs and quitting intent. Tob Control.

[B27] Rodu B, Cole P (2002). Smokeless tobacco use and cancer of the upper respiratory tract. Oral Surg Oral Med Oral Pathol Oral Radiol Endod.

[B28] Phillips CV, Wang C, Guenzel B (2005). You might as well smoke; the misleading and harmful public message about smokeless tobacco. BMC Public Health.

[B29] Accortt NA, Waterbor JB, Beall C, Howard G (2005). Cancer incidence among a cohort of smokeless tobacco users (United States). Cancer Causes Control.

[B30] Asplund K (2003). Smokeless tobacco and cardiovascular disease. Prog Cardiovasc Dis.

[B31] Hirschhorn N, Biialous SA, Shachtenstein S (2001). Philip Morris'new scientific initiative: an analysis. Tob Control.

[B32] World Bank (1999). Curbing the epidemic: Governments and the economics of tobacco control. Development in practice series.

[B33] Tomar SL (2003). Smokeless tobacco use is a significant predictor of smoking when appropriately modeled. Nicotine Tob Res.

[B34] Buck DJ, Richmond R, Mendelsohn CP (2000). Cost-effectiveness analysis of a family physician delivered smoking cessation program. Prev Med.

[B35] Hughes JR, Carpenter MJ (2005). The feasibility of smoking reduction: an update. Addiction.

[B36] Fagerström KO, Schildt EB (2003). Should the European union lift the ban on snus? Evidence from the Swedish experience. Addiction.

[B37] Hatsukami DK, Benowitz NL, Rennard SI, Oncken C, Hecht SS (2006). Biomarkers to assess the utility of potential reduced exposure tobacco products. Nicotine Tob Res.

